# Survival Impact of Long-Term Tramadol Use on Breast Cancer for Patients with Chronic Pain: A Propensity Score-Matched Population-Based Cohort Study

**DOI:** 10.3390/jpm12030384

**Published:** 2022-03-02

**Authors:** Mingyang Sun, Chia-Lun Chang, Chang-Yun Lu, Szu-Yuan Wu, Jiaqiang Zhang

**Affiliations:** 1 Department of Anesthesiology and Perioperative Medicine, People’s Hospital of Zhengzhou University, Henan Provincial People’s Hospital, Zhengzhou 450052, China; esther0613@tmu.edu.tw; 2 Department of Hemato-Oncology, Wan Fang Hospital, Taipei Medical University, Taipei 110, Taiwan; richardch9@hotmail.com; 3 Department of Internal Medicine, School of Medicine, College of Medicine, Taipei Medical University, Taipei 110, Taiwan; 4 Department of General Surgery, Lo-Hsu Medical Foundation, Lotung Poh-Ai Hospital, Yilan 265, Taiwan; b8301101lupin@yahoo.com.tw; 5 Department of Food Nutrition and Health Biotechnology, College of Medical and Health Science, Asia University, Taichung 413, Taiwan; 6 Big Data Center, Lo-Hsu Medical Foundation, Lotung Poh-Ai Hospital, Yilan 265, Taiwan; 7 Division of Radiation Oncology, Lo-Hsu Medical Foundation, Lotung Poh-Ai Hospital, Yilan 265, Taiwan; 8 Department of Healthcare Administration, College of Medical and Health Science, Asia University, Taichung 413, Taiwan; 9 Graduate Institute of Business Administration, College of Management, Fu Jen Catholic University, Taipei 242062, Taiwan; 10 Centers for Regional Anesthesia and Pain Medicine, Wan Fang Hospital, Taipei Medical University, Taipei 110, Taiwan

**Keywords:** tramadol, analgesia, breast cancer, survival, prognosis

## Abstract

Purpose: The impact of tramadol analgesic use before breast cancer diagnosis on survival in patients with chronic pain is unclear. Therefore, we designed a propensity score-matched population-based cohort study to compare the breast cancer-related survival of patients with chronic pain who received long-term tramadol analgesic treatment with that of those who did not receive such treatment. Patients and Methods: We included patients with chronic pain and categorized them into two groups according to their analgesic use, comparing their breast cancer-related survival; patients with breast cancer and chronic pain who were prescribed ≥180 defined daily doses (DDDs) of tramadol analgesics per year >3 months before breast cancer diagnosis comprised the case group, and those who were prescribed non-tramadol analgesics before breast cancer diagnosis comprised the control group. Patients in both groups were matched at a ratio of 1:5. Results: The matching process yielded a final cohort of 624 patients (104 and 520 in the case and control groups, respectively) who were eligible for further analysis. According to both univariate and multivariate Cox regression analyses, the adjusted hazard ratio for all-cause death in the case group compared with in the control group was 3.45 (95% confidence interval = 2.36–5.04; *p* < 0.001). Conclusion: Long-term tramadol analgesic use prior to breast cancer diagnosis might be associated with poor overall survival in patients with chronic pain compared with such patients that did not receive long-term tramadol analgesic treatment.

## 1. Introduction

Chronic pain is one of the most common reasons that patients seek medical attention [1]. Chronic pain results from combined biologic, psychologic, and social factors, and most often requires a multifactorial approach to management [1]. The treatment of chronic pain begins with and should always include nonpharmacological approaches [2]. For patients with inadequate analgesia despite nonpharmacologic therapies, physicians add carefully selected multitargeted pharmacological therapies, based on the type of pain [3]. Tramadol, which is mixed mechanism opioids with additional norepinephrine and serotonin reuptake inhibition, are of uncertain benefit for patients with chronic pain [4,5]. Tramadol may be considered as second or third line treatment for patients with chronic pain such as fibromyalgia who have not responded to initial therapy with other medications. However, these opioid medications have the same risks associated with all opioids and must be used judiciously and carefully [6]. 

There have been some conflicting data of tramadol use for non-cancer pain and cancer pain and all-cause mortality [7,8,9,10,11,12,13,14,15,16]. Recently, one study showed tramadol prescription may be associated with increased all-cause mortality compared with commonly prescribed nonsteroidal anti-inflammatory drugs (NASIDs) for patients with osteoarthritis [9]. Another study demonstrated that new prescription dispensation of tramadol, compared with codeine, was significantly associated with a higher risk of mortality, cardiovascular events, and fracture [8]. However, a positive study showed that tramadol use is associated with enhanced postoperative oncologic outcomes of survival and recurrence in breast cancer patients with a retrospective clinical study and in vitro confirmation [7]. The previous data demonstrated the anti-breast cancer effect in preclinical and clinical studies [7,10]. The anti-breast cancer effect of short-term tramadol use [7,10] and the safety of long-term tramadol use [8,9] both raised our interest recently. The different duration, dose, and follow-up time of tramadol use in non-cancer pain patients or cancer pain patients might be contributed to different survival outcomes resulting in controversial reports [7,8,9].

Therefore, we conducted a head-to-head propensity score matching (PSM) study to clarify the tramadol use for non-cancer pain patients before cancer diagnosis. Our study could clarify the influence of survival in long-term tramadol analgesic users and nonusers for non-cancer pain patients who would have breast cancers in the future. The long-term tramadol analgesic use for chronic pain patients before breast cancer diagnosis might be associated with survival impact in breast cancer patients based on the previous conflicting data [7,8,9,10]. Understanding the survival impact of long-term tramadol analgesic use and nonuse for chronic pain patients before breast cancer diagnosis could be a good reference for decision-making sharing for analgesics between physicians and patients with chronic pain.

## 2. Patients and Methods

### 2.1. Data Sources and Study Cohort

From the Taiwan Cancer Registry database (TCRD), we identified patients with chronic pain who had received a diagnosis of breast cancer between 1 January 2008, and 31 December 2017. The index date was the date of breast cancer diagnosis. The follow-up duration was from the index date to 31 December 2019. The study protocols were reviewed and approved by the Institutional Review Board of Tzu-Chi Medical Foundation (IRB109-015-B). Data from the cancer registry database of the Collaboration Center of Health Information Application, which contains detailed cancer-related information regarding pathological types, cancer stages, and treatments, were also used [17,18,19,20].

### 2.2. Participant Selection

Inclusion criteria were a diagnosis of chronic pain, aged >20 years, receiving analgesics, and having breast cancer without metastasis. Chronic pain was defined as originating from one of the following: osteoarthritis (International Classification of Diseases, Ninth Revision, Clinical Modification (ICD-9-CM): 715), spinal disorders (ICD-9-CM: 756.11, 756.12, 720–725, 737.1–737.4), peripheral vascular diseases (ICD-9-CM: 443.8–444.9), osteoporosis (ICD-9-CM: 733.0), gout (ICD-9-CM: 274), headache (ICD-9-CM: 307.81, 784.0, 346), diabetic neuropathy (ICD-9-CM: 250.6, 357.2), rheumatoid arthritis (ICD-9-CM: 714), pressure ulcer (ICD-9-CM: 707), or herpes zoster (ICD-9-CM: 053). The exclusion criterion was a history of cancer before diagnosis of breast cancer. Long-term tramadol analgesia use was defined as use of tramadol on most days for >3 months with a mean tramadol dose of ≥180 defined daily doses (DDDs) per year [21,22]. Patients with chronic pain who were prescribed ≥180 DDDs of tramadol analgesics annually with a prescription duration of >3 months before the index date comprised the case group and those who were prescribed no tramadol analgesics use before the index date comprised the control group. Non-tramadol analgesia use for chronic pain patients could be NSAIDs or other opioid drug use. Tramadol analgesic use after breast cancer diagnosis was not considered during enrollment for prevention of the bias that the severity of cancer pain and breast cancer severity contributed to all-cause mortality. The endpoint was all-cause death in these chronic pain patients with breast cancer who received long-term tramadol analgesic use (versus the controls) before the index date.

### 2.3. Propensity Scores Matching and Covariates

After adjustment for confounders, we used a time-dependent Cox proportional hazards model to model the time from the index date to all-cause death for patients with chronic pain and breast cancer. To reduce the effects of potential confounders when comparing all-cause death between the long-term tramadol analgesic user and nonuser groups before breast cancer treatments, participants were matched based on propensity scores. The matching variables used were sex, age, comorbidities, income levels, urbanization, menopausal status, Human Epidermal Growth factor Receptor-2 (HER2) status, nodal surgical types (sentinel lymph node biopsy or axillary lymph node dissection), American Joint Committee on Cancer (AJCC) clinical stage, hormone receptor status, breast surgical types (breast conservation or total mastectomy), differentiation of tumor, chemotherapy, and adjuvant radiotherapy. Definition of clinical cancer stages in our study was based on the clinical AJCC, 7th Edition for breast cancer stages. Comorbidities were determined according to ICD-9-CM codes in the main diagnosis of inpatient records or if the number of outpatient visits was ≥2 within 1 year. Comorbidities onset at 12 months before the index date were recorded. Continuous variables are presented as means ± standard deviations or medians (first quartile, third quartile), where appropriate. Hormone receptor positivity was defined as ≥1% of tumor cells demonstrating positive nuclear staining through immunohistochemistry [23], and HER2 positivity was defined as an immunohistochemistry score of 3+ or a fluorescence in situ hybridization ratio of ≥2 [24,25]. We matched participants at a ratio of 1:5 by using the greedy method, with sex, age, comorbidities, income levels, urbanization, menopausal status, HER2 status, nodal surgical types, AJCC clinical stage, hormone receptor status, breast surgical types, differentiation of tumor, chemotherapy, and adjuvant radiotherapy matched with a propensity score within a caliper of 0.2 [26]. Matching is a common technique used for selecting controls with identical background covariates as study participants to minimize differences among study participants (that the investigator deems necessary to be controlled). A Cox model was used to perform regression on the variable of all-cause death in long-term tramadol analgesic users and nonusers before breast cancer diagnosis, and a robust sandwich estimator was used to account for the clustering within matched sets [27]. Multivariate Cox regression analysis was performed to calculate hazard ratios (HRs) with 95% confidence interval (CI) to determine whether factors such as tramadol use, sex, age, comorbidities, income levels, urbanization, menopausal status, HER2 status, nodal surgical types, AJCC clinical stage, hormone receptor status, breast surgical types, differentiation of tumor, chemotherapy, and adjuvant radiotherapy were potential independent predictors of all-cause death.

### 2.4. Statistical Analysis

All analyses were performed using SAS version 9.3 (SAS Institute, Cary, NC, USA). In a two-tailed Wald test, *p* < 0.05 was considered significant. Overall survival (OS) was estimated using the Kaplan–Meier method, and differences between the long-term tramadol users and nonusers were determined using the stratified log-rank test to compare survival curves (stratified according to matched sets) [28].

## 3. Results

### 3.1. Study Cohort

The matching process yielded a final cohort of 624 patients (520 and 104 in the long-term tramadol analgesic user and nonuser groups, respectively) who were eligible for further analysis; their characteristics are summarized in Table 1. Age distribution was balanced between the two groups (Table 1). Sex, age, comorbidities, income levels, urbanization, menopausal status, HER2 status, nodal surgical types, AJCC clinical stage, hormone receptor status, breast surgical types, differentiation of tumor, chemotherapy, and adjuvant radiotherapy were similar after head-to-head PSM of the two groups, with no significant differences in the variables observed between the groups. In broad terms, the primary endpoint of all-cause death in patients with chronic pain with long-term tramadol analgesic use (before breast cancer diagnosis) varied significantly from the control group (*p* < 0.001; Table 1).

### 3.2. All-Cause Death after Multivariate Cox Regression Analysis

**Figure 1:**
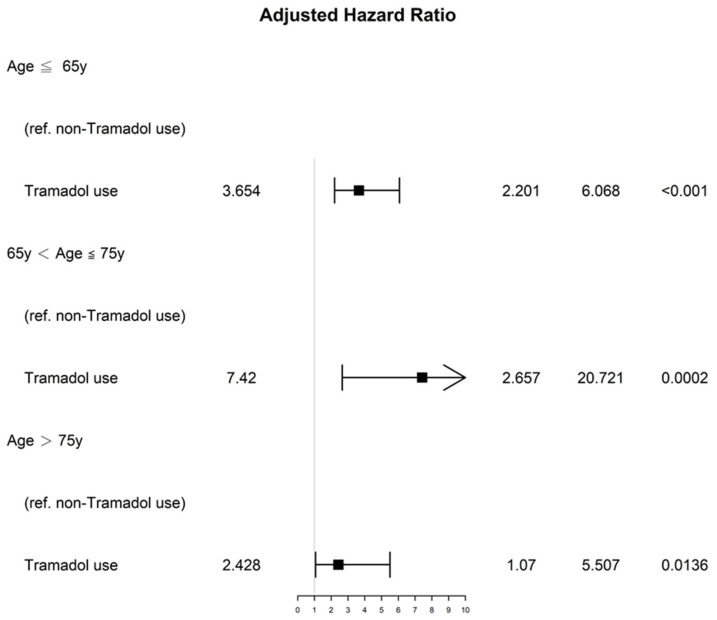
Forest plots of the adjusted hazards ratios for all-cause death in patients who received long-term tramadol analgesic therapy before breast cancer diagnosis compared with those who did not, stratified by age groups.

The results of multivariate Cox regression analysis indicated that the group that used tramadol analgesics before breast cancer diagnosis exhibited less favorable prognostic factors for OS (Table 2). No significant differences were observed in the explanatory variables, except for older age. In the multivariate Cox regression analysis, the aHR (95% CI) of all-cause death for the long-term tramadol analgesic users before breast cancer diagnosis compared with that of the control group was 3.45 (2.36–5.04; *p* < 0.001). The aHRs (95% CIs) of all-cause death for those aged 66 to 75 years, 76 to 85 years, and >85 years (compared with age ≤ 65 years) were 1.6 (1.03–2.46), 2.6 (1.63–4.17), and 5.09 (2.33–11.12), respectively (Table 2). A stratified analysis of the distinct age groups are presented as a forest plot in Figure 1. The aHRs (95% CIs) for patients with chronic pain and breast cancer who used long-term tramadol analgesics before breast cancer diagnosis were significantly associated with higher mortality compared with the control group, regardless of age groups.

### 3.3. Kaplan–Meier Survival Curve of Long-Term Tramadol Analgesic Users and Nonusers before Breast Cancer Diagnosis

**Figure 2:**
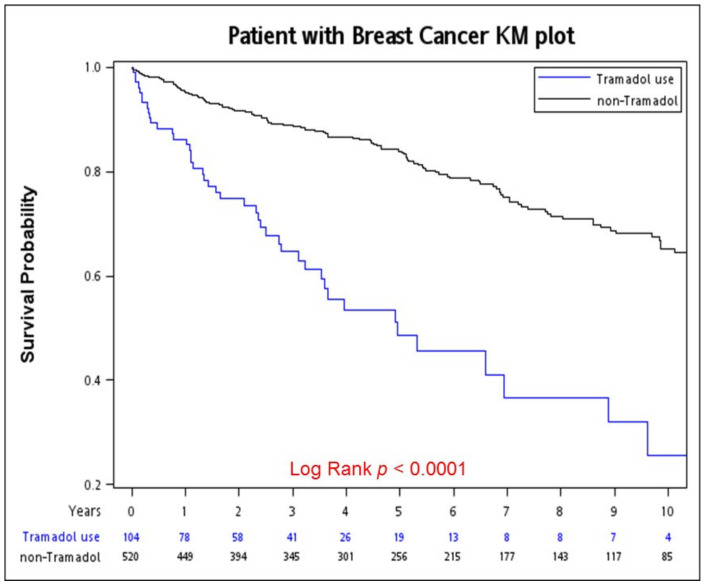
Kaplan–Meier overall survival curves of propensity scores matching chronic pain patients using tramadol or non-tramadol analgesic drugs before breast cancer diagnosis.

Figure 2 presents the survival curve (in terms of OS) obtained using the Kaplan–Meier method for the PSM cohort of patients with chronic pain who either received or did not receive long-term tramadol analgesic treatment before breast cancer diagnosis. The 5-year OS curves for the long-term tramadol analgesic users and nonusers were 82.92% and 52.42%, respectively (*p* < 0.001).

## 4. Discussion

Tramadol is a mixed mechanism opioid with weak affinity to the mu opioid receptor and serotonin and norepinephrine reuptake inhibition [4,5]. Similar to other opioids, it may be used as a second-line agent for patients with chronic pain who have not responded to initial therapy with other agents [6]. There were some studies showing that tramadol is reported to promote or preserve immunity including natural killer (NK) cell activity, which is important in anti-cancer defenses [1,7,10]. Preclinical studies have shown that this is not a class effect, in that morphine and fentanyl suppress NK cell cytotoxicity; buprenorphine does not affect NK cell cytotoxicity, whereas tramadol increases NK cell cytotoxicity, reducing metastasis [7,10,16]. After breast cancer surgery, patients who received tramadol had a decreased risk of postoperative recurrence and mortality [7]. The anti-breast cancer effect of tramadol appears to involve inhibition of proliferation, induction of apoptosis, and effects on 5-HT_2B_ receptor and TRPV-1 [7]. Nevertheless, there was an increased mortality risk associated with tramadol in the adult population noted in the other previous studies [8,9]. Although tramadol is reported to be effective in pain management, its long-term toxicity should be kept in mind.

Our PSM cohort study was balanced between case and control groups (Table 1), showing a similar model to the randomized controlled trial (RCT) [29]. PSM enables the design of an observational (nonrandomized) study that shares several key RCT characteristics [29]. There were no significant differences between case and control groups of cofounding factors in Table 1 that might be associated with the endpoint. Therefore, there were few selection biases between long-term tramadol analgesic use and non-tramadol use before breast cancer diagnosis, although there may have been some confounding factors not accounted for in the model associated with all-cause death. Until now, our study is the first comparative PSM study to mimic an RCT to estimate the survival effect of chronic pain patients with long-term tramadol analgesic use before breast cancer diagnosis. All confounding factors associated with breast cancer survival have been matched between the case and control groups [17,20,30,31]. The crude all-cause death in the PSM chronic pain patients with long-term tramadol analgesic use before breast cancer was higher than non-tramadol users (Table 1). Our findings showed long-term tramadol analgesic use before breast cancer might be harmful for OS in breast cancer, which was different from the previous study [7]. Kim et al. demonstrated that tramadol after breast cancer surgery had a decreased risk of postoperative mortality. The anti-breast cancer effect of tramadol appears to involve the inhibition of proliferation, induction of apoptosis, and effects on 5-HT2B receptor and TRPV-1 for Michigan Cancer Foundation-7 (MCF-7, breast adenocarcinoma cell) [7]. MCF-7 cells are commonly used in research for estrogen receptor positive, progesterone receptor positive, and HER2 negative breast cancer cell experiments [32]. However, women with breast cancer types were heterogeneous types, instead of only the hormone receptor positive type [33]. The potential different survival outcomes of tramadol use in breast cancer might be due to the different durations and time-points of tramadol use (before or after breast cancer diagnosis) between ours and Kim et al.’s study [7]. In other studies, Zeng et al. and Xie et al. showed longer tramadol use for non-cancer chronic pain was associated with a significantly higher rate of mortality over years of follow-up compared with commonly prescribed non-tramadol analgesics [8,9]. The conflicting data among Kim’s, Zeng’s, and Xie’s studies might also be contributed to by the duration or time-point of tramadol use (before or after cancer diagnosis) [7,8,9]. Our duration and time-point of tramadol use (before or after breast cancer diagnosis) were similar to Zeng’s and Xie’s studies having compatible outcomes for which long-term tramadol use before breast cancer diagnosis might be associated with higher mortality [8,9].

The mechanism of tramadol use for increased or decreased mortality is still unclear. Although Kim et al.’s study showed postoperative analgesics of tramadol use in breast cancer patients could decrease recurrence and mortality compared to other analgesic drugs, the tramadol use was short-term, instead of long-term use [7]. In addition, the duration of tramadol use was only 24 h with MCF-7 cells to evaluate the anti-cancer effects with concentrations ranging from 0.05 to 2.5 mg mL^−1^ in vitro experiments [7]. The different concentration and duration of tramadol use in human beings might result in different effects in oncologic outcomes. Unlike morphine, tramadol did not suppress cellular immune function but did increase the activity of NK cells, proliferation of lymphocytes, and production of interleukin-2 [16]. Another study showed that tramadol activates the serotoninergic system and may have a positive effect on the immune system by increasing proliferation of lymphocytes and NK cell activity [10]. In fact, the previous studies showed the anti-cancer effects of tramadol in short-term (hours, during perioperative or postoperative days) use [7,10,16], with only a few studies indicating that long-term tramadol use is beneficial or safe for oncologic outcomes. By contrast, increased mortality has been noted in studies investigating long-term tramadol use [8,9]. The biological mechanisms linking long-term tramadol use to mortality are unclear. Long-term tramadol use may activate μ opioid receptors and inhibit central serotonin and norepinephrine reuptake, and the latter may result in unique adverse effects on the neurological system such as serotonin syndrome and seizures [34]. Tramadol might also increase the risk of postoperative delirium, which tends to increase mortality [35]. Fatal poisoning or respiratory depression may occur when long-term tramadol users consume alcohol or use tramadol with other central nervous systems depressants [36,37,38,39]. Furthermore, tramadol may increase the risk of hypoglycemia, hyponatremia, fracture, or fall, thus leading to an increased risk of death [40,41,42,43]. Few studies have the definition of “long-term” tramadol use for chronic pain. However, in our study, long-term tramadol analgesia use was defined as use of tramadol on most days for >3 months with a mean tramadol dose of ≥180 DDDs per year. The potential reasons for long-term tramadol use associated with an increase in mortality in breast cancer might be attributed to the aforementioned causes.

Although all the covariates were matched through PSM, a residual imbalance in age between the groups contributed to older age being a significant prognostic factor of poor OS after the multivariate Cox regression analysis (Table 2) [44,45]. We obtained aHRs for all-cause death in the case and control groups, stratified by age (Figure 1). After stratified analysis of age groups, long-term tramadol use before breast cancer diagnosis is an independent risk of all-cause death, whatever the age group. Our study is the first head-to-head PSM with the longest follow-up to evaluate the survival impact of long-term tramadol use before breast cancer diagnosis. Our study showed chronic pain patients with breast cancer having long-term tramadol analgesic use before breast cancer diagnosis were associated with poor OS compared with those having non-tramadol analgesic use. Up to now, there has been no sufficient sample size or follow-up time to evaluate the impact of long-term tramadol analgesic use on survival for chronic pain patients with breast cancer, thus, ours is the first study to do this. In general, based on results reported in the current study, non-tramadol analgesic use could be preferred for the management of chronic pain. In addition, long-term tramadol analgesic use for chronic pain patients with a high risk of breast cancer in the future (breast cancer family history, White race, obesity, early menarche, later age at the time of first pregnancy, absence of breastfeeding, nulliparity, estrogen/progesterone menopausal hormone therapy, alcohol use, and current smoking, etc.) should be identified and patients informed of the risk of poor OS in breast cancer because non-tramadol analgesic use might be associated with better survival for breast cancer patients compared with tramadol use.

The strength of our study is that it is the first long-term follow-up cohort study to estimate the primary endpoint of OS for patients with chronic pain receiving tramadol analgesia and non-tramadol analgesia before they received a breast cancer diagnosis. The covariates between the groups in the study were homogenous for patients with chronic pain, and no selection bias was identified through PSM (Table 1). No other studies have assessed the impact of long-term tramadol analgesia use and non-tramadol analgesia use by patients with chronic pain who subsequently received a diagnosis of breast cancer. Our study revealed that long-term tramadol analgesia use is associated with increased risk of all-cause death in patients with breast cancer. Although some studies have demonstrated the survival benefits of postoperative short-term tramadol use for patients with breast cancer, physicians should consider the risk of mortality when prescribing long-term tramadol analgesic use for chronic pain, especially in patients at a high risk of breast cancer. (Figure 1).

This study has some limitations. First, because all patients with cancer were enrolled from an Asian population, the corresponding ethnic susceptibility compared with the non-Asian population remains unclear; hence, our results should be cautiously extrapolated to non-Asian populations. However, there is no evidence to demonstrate significant differences in oncologic outcomes for breast cancer survivors between Asian and non-Asian patients. Second, the diagnoses of all comorbid conditions were based on ICD-9-CM codes. Nevertheless, the Taiwan Cancer Registry Administration randomly reviews charts and interviews patients to verify the accuracy of the diagnoses, and hospitals with outlier chargers or practices may be audited and, subsequently, heavily penalized if malpractice or discrepancies are identified. Accordingly, to obtain crucial information on population specificity and disease occurrence, a large-scale randomized trial comparing carefully selected patients undergoing tramadol analgesia use and non-tramadol analgesia use before breast cancer diagnosis should be carried out. Nevertheless, it is very difficult to perform this kind of RTC to estimate the effects of tramadol use for patients without breast cancer. Finally, the TCRD does not contain information regarding dietary habits or body mass index, all of which may be risk factors for OS. Despite these limitations, a major strength of this study is the use of a nationwide population-based registry with detailed baseline information. Lifelong follow-up was possible through the linkage of the registry with the national Cause of Death database. Considering the magnitude and statistical significance of the observed effects in the current study, the limitations are unlikely to affect our conclusions.

## 5. Conclusions

Long-term tramadol analgesia use before breast cancer diagnosis might be associated with a reduction in OS for patients with chronic pain compared with non-tramadol analgesia use. Although some studies have demonstrated the survival benefits of postoperative short-term tramadol use for patients with breast cancer, physicians should consider the risk of mortality when prescribing long-term tramadol analgesic use for chronic pain, especially in patients at a high risk of breast cancer.

## Figures and Tables

**Table 1:** Tramadol or non-tramadol analgesic drugs use for chronic pain patients before breast cancer diagnosis after propensity scores matching.

	Tramadol Analgesia		Non-Tramadol Analgesia		*p*-Value
	N = 520		N = 104		
	N	%	N	%	
Sex					0.8103
Female	492	94.62%	99	95.19%	
Male	28	5.38%	5	4.81%	
Age (mean ± SD)	59.36 ± 13.03		59.83 ± 13.68		0.7405
Age (years old)					0.1211
Age ≤ 65	351	67.50%	67	64.42%	
65y < Age ≤ 75	98	18.85%	16	15.38%	
75 < Age ≤ 85	54	10.38%	19	18.27%	
Age>85	17	3.27%	2	1.92%	
Comorbidities					
CCI Score (mean ± SD)	0.51 ± 0.95		0.52 ± 0.97		0.9403
CCI Score					0.9681
=0	376	72.31%	75	72.12%	
≥1	144	27.69%	29	27.88%	
Congestive Heart Failure	21	4.04%	7	6.73%	0.2260
Dementia	9	1.73%	3	2.88%	0.4341
Chronic Pulmonary Disease	59	11.35%	11	10.58%	0.8205
Rheumatic Disease	12	2.31%	2	1.92%	0.8090
Hepatitis B/C	53	10.19%	7	6.73%	0.2743
Diabetes with complications	17	3.27%	2	1.92%	0.4658
Hemiplegia and Paraplegia	0	0.00%	0	0.00%	-
Renal Disease	10	1.92%	3	2.88%	0.5308
AIDS	0	0.00%	0	0.00%	-
Diabetes	79	15.19%	16	15.38%	0.9603
Hyperlipidemia	98	18.85%	18	17.31%	0.7128
ESRD	0	0.00%	0	0.00%	-
Liver cirrhosis	83	15.96%	17	16.35%	0.9222
AMI	10	1.92%	2	1.92%	1.0000
Coronary Arterial Disease	55	10.58%	13	12.50%	0.5656
Hemorrhage Stroke	13	2.50%	2	1.92%	0.7259
Ischemia Stroke	10	1.92%	2	1.92%	1.0000
Income levels (NTD/month)					0.9765
Low income	10	1.92%	2	1.92%	
Income ≤ 20,000	258	49.62%	54	51.92%	
20,000 < Income ≤ 30,000	176	33.85%	34	32.69%	
Income > 30,000	76	14.62%	14	13.46%	
Urbanization					0.6248
Rural	104	20.00%	23	22.12%	
Urban	416	80.00%	81	77.88%	
Menopausal status					0.914
Postmenopausal	338	65.00%	67	64.42%	
Premenopausal	182	35.00%	37	35.58%	
HER2 status					1.000
Negative	415	79.81%	83	79.81%	
Positive	105	20.19%	21	20.19%	
Nodal surgery					1.000
SLNB	360	69.23%	72	69.23%	
ALND	160	30.77%	32	30.77%	
AJCC clinical stage					1.000
I	270	51.92%	54	51.92%	
II	130	25.00%	26	25.00%	
III	120	23.08%	24	23.08%	
Hormone receptor					1.000
Negative	120	23.08%	24	23.08%	
Positive	400	76.92%	80	76.92%	
Breast surgery					1.000
Total mastectomy	85	16.35%	17	16.35%	
Breast-conserving surgery	435	83.65%	87	83.65%	
Differentiation					1.000
I	80	15.38%	16	15.38%	
II	230	44.23%	46	44.23%	
III	210	40.38%	42	40.38%	
Chemotherapy					0.898
No	273	52.50%	54	51.92%	
Yes	247	47.50%	50	48.08%	
Adjuvant radiotherapy					0.834
No	87	16.73%	17	16.35%	
Yes	433	83.27%	87	83.65%	
Follow-up time, Years, (mean ± SD)	5.78 ± 4.45		3.12 ± 3.20		<0.0001
All-cause Death					<0.0001
Not	397	76.35%	60	57.69%	
Yes	123	23.65%	44	42.31%	

AIDS, acquired immune deficiency syndrome; AMI, acute myocardial infarction; CAD, coronary artery disease; CCI, Charlson comorbidity index; ESRD, end stage renal disease; IQR, interquartile range; SD, standard deviation; SLNB, sentinel lymph node biopsy; ALND, axillary lymph node dissection; NTD, New Taiwan dollar; AJCC, American Joint Committee on Cancer; HER2, Human Epidermal Growth factor Receptor-2.

**Table 2:** Cox proportional models of all-cause death for propensity scores matching chronic pain patients with breast cancer.

	Crude HR (95% CI)		Adjusted HR * (95% CI)		*p*-Value
Tramadol Analgesia (ref. Non-Tramadol Analgesia)					
Tramadol use	3.33	(2.34–4.75)	3.45	(2.36–5.04)	<0.001
Sex (ref. female)					
male	2.76	(1.71–4.46)	1.34	(0.74–2.41)	0.3310
Age (ref. Age ≤ 65 years old)					
65 < Age ≤ 75	1.61	(1.09–2.38)	1.6	(1.03–2.46)	0.035
75 < Age ≤ 85	2.51	(1.66–3.78)	2.6	(1.63–4.17)	<0.001
Age > 85	5.86	(3.18–10.78)	5.09	(2.33–11.12)	<0.001
Comorbidities					
Congestive Heart Failure (ref. no)	1.44	(0.90–2.30)	1.07	(0.94–1.77)	0.913
Dementia (ref. no)	2.01	(0.86–4.11)	1.26	(0.49–2.83)	0.657
Chronic Pulmonary Disease (ref. no)	1.03	(0.77–1.40)	1.01	(0.74,1.39)	0.642
Rheumatic Disease (ref. no)	1.08	(0.81–2.17)	1.05	(0.90–2.05)	0.341
Hepatitis B/C (ref. no)	1.06	(0.89–2.02)	1.02	(0.91–1.86)	0.410
Diabetes with complications (Severe diabetes) (ref. no)	1.05	(0.59–3.21)	1.03	(0.69–2.77)	0.538
Renal Disease (ref. no)	1.14	(0.91–2.00)	1.08	(0.81–1.81)	0.308
Diabetes (ref. no)	1.77	(1.2–2.6)	1.48	(0.92–2.36)	0.104
Hyperlipidemia (ref. no)	1.05	(0.7–1.59)	1.09	(0.54–1.49)	0.683
Liver cirrhosis (ref. no)	1.00	(0.64–1.56)	0.75	(0.45–1.25)	0.272
AMI (ref. no)	2.15	(0.88–5.25)	1.38	(0.48–3.92)	0.551
Coronary Arterial Disease (ref. no)	1.51	(0.95–2.39)	0.97	(0.54–1.73)	0.909
Hemorrhage Stroke (ref. no)	2.08	(0.92–4.7)	2.05	(0.82–5.15)	0.126
Ischemia Stroke (ref. no)	1.46	(0.46–4.57)	1.03	(0.26–3.31)	0.912
Urbanization (ref. Rural)					
Urban	0.74	(0.52–1.05)	0.76	(0.52–1.12)	0.161
Income levels (ref. low income, NTD/month)					
Income ≤ 20,000	0.50	(0.23–1.07)	0.71	(0.31–1.65)	0.425
20,000 < Income ≤ 30,000	0.30	(0.14–0.68)	0.54	(0.22–1.3)	0.170
Income > 30,000	0.26	(0.11–0.63)	0.49	(0.18–1.3)	0.152
Menopausal status (ref: Postmenopausal)					
Premenopausal	1.11	(0.98–1.71)	1.10	(0.90–1.21)	0.317
HER2 (ref: Negative)					
Positive	1.49	(1.01–1.70)	1.19	(0.84–1.10)	0.488
Breast surgery (ref: Total mastectomy)					
Breast-conserving surgery	1.09	(0.80–1.15)	1.02	(0.79–1.09)	0.473
Nodal surgery (ref: SLND)					
ALND	1.11	(0.66–1.33)	1.06	(0.68–1.27)	0.501
AJCC clinical stage (ref. stage I)					
Stage II	1.21	(0.93–2.16)	1.20	(0.96–1.90)	0.081
Stage III	1.91	(0.69–2.01)	1.59	(0.83–1.81)	0.094
Hormone receptor (ref. Negative)					
Positive	0.88	(0.79–1.38)	0.93	(0.87–1.29)	0.441
Differentiation (ref: Grade I)					
Grade II	1.06	(0.92–1.16)	1.03	(0.91–1.17)	0.061
Grade III	1.09	(0.94–1.18)	1.08	(0.97–1.15)	0.073
Chemotherapy (ref: No)					
Yes	0.71	(0.51–1.09)	0.82	(0.62–1.10)	0.524
Adjuvant radiotherapy (ref: No)					
Yes	0.88	(0.61–1.20)	0.89	(0.62–1.19)	0.415

aHR, adjusted hazard ratio; AIDS, acquired immune deficiency syndrome; AMI, acute myocardial infarction; CCI, Charlson comorbidity index; CI, confidence interval; DDD, defined daily dose; HR, hazard ratio; ref., reference group; SLNB, sentinel lymph node biopsy; ALND, axillary lymph node dissection; NTD, New Taiwan dollar; AJCC, American Joint Committee on Cancer; HER2, Human Epidermal Growth factor Receptor-2. * All covariates presented in Table 2 were adjusted.

## Data Availability

The datasets supporting the study conclusions are included within this manuscript and its additional files. For Software: Project name: not applicable; project homepage: not applicable; archived version: not applicable; operating system(s): not applicable; programming language: not applicable; other requirements: not applicable; license: not applicable; any restrictions to use by nonacademicians: not applicable.

## Abbreviations

DDD	Defined Daily Dose
HR	hazard ratio
aHR	adjusted hazard ratio
CI	confidence interval
RCT	randomized controlled trial
PSM	propensity score matching
TCRD	Taiwan Cancer Registry database
ICD-9-CM	International Classification of Diseases, Ninth Revision, Clinical Modification
OS	overall survival
CCI	Charlson comorbidity index
NK	natural killer
NSAID	nonsteroidal anti-inflammatory drugs
AJCC	American Joint Committee on Cancer
HER2	Human Epidermal Growth factor Receptor-2
MCF-7	Michigan Cancer Foundation-7
